# Structure-Based Prototype Peptides Targeting the *Pseudomonas aeruginosa* Type VI Secretion System Effector as a Novel Antibacterial Strategy

**DOI:** 10.3389/fcimb.2017.00411

**Published:** 2017-09-20

**Authors:** Xiaopan Gao, Zhixia Mu, Bo Qin, Yicheng Sun, Sheng Cui

**Affiliations:** MOH Key Laboratory of Systems Biology of Pathogens, Institute of Pathogen Biology, Chinese Academy of Medical Sciences and Peking Union Medical College Beijing, China

**Keywords:** *Pseudomonas aeruginosa*, T6SS, effector-immunity, TplE-TplEi interaction, toxin-antitoxin, antibacterial peptide

## Abstract

The type VI secretion system (T6SS) secretes numerous toxins for bacteria-bacteria competition. TplE is a newly identified trans-kingdom toxin secreted by the T6SS in *Pseudomonas aeruginosa*, while TplEi neutralizes the toxic effect of TplE to protect bacteria autointoxication. Blocking the interaction of TplE-TplEi could unleash the toxin, causing bacterial cell death. In this study, we applied a crystallographic approach to design a structural-based antimicrobial peptides targeting the interaction of TplE and TplEi. We found that a peptide (designed as “L” peptide based on its shape) derived from TplE can form a crystal complex with TplEi after subtilisin treatment and the crystal structure was solved at 2.2Å. The “L” peptide displays strong binding affinity to TplEi *in vitro* and can release the TplE toxin to induce bacteria death *in vivo*. Our findings suggest that as a toxin activator, the “L” peptide could be a possible drug lead for treating *P. aeruginosa* infection. Our findings provide an example that the T6SS effector and immunity protein could be a potential drug target against bacteria infection.

## Introduction

*Pseudomonas aeruginosa* is one of the most common nosocomial infectious bacteria that causes significant morbidity and mortality in immunocompromised patients or intensive care unit (ICU) patients (Oliver et al., 2015). *P. aeruginosa* strains has developed multidrug-resistant (MDR) or even extensively drug-resistant (XDR) phenotypes, which are resistant to most of the currently used antibiotics. Thus, it is an urgent need to develop novel effective antibacterial agents (Reardon, 2014).

The type VI secretion system (T6SSs) has recently garnered more attention than ever because of its widespread occurrence and significance in the ecosystem function and human health (Russell et al., 2014). T6SS is a versatile molecular machine deployed by many bacterial species to deliver protein effectors into both prokaryotic and eukaryotic cells. T6SS is typically encoded in large and variable gene clusters via 14 conserved “core” proteins essential for function and a variable complement of accessory elements (Shneider et al., 2013; Cianfanelli et al., 2016). Bioinformatics and structural evidence have shown that T6SS is believed to resembles a bacteriophage tail-like structure, and the contraction of this device delivers multiple, diverse effector proteins directly into the recipient cells in a dynamic “firing” mechanism (Cianfanelli et al., 2016). The “firing” mechanism takes two forms. In the first form, the effectors are fused to structural components (“specialized” effectors) such as PAAR and VgrG proteins. In the second form, there is a noncovalent interaction with one of the core components (“cargo” effectors), such as Hcp1 and Tse2 of the H1-T6SS of *P. aeruginosa* (Silverman et al., 2013; Durand et al., 2014; Whitney et al., 2014).

*P. aeruginosa* encodes three distinct T6SS hemolysin coregulated protein (Hcp) secretion islands (named H1-to H3-T6SS) that act against both prokaryotic and eukaryotic cells (Shneider et al., 2013; Cianfanelli et al., 2016; Sana et al., 2016). Recently, considerable progress has been made in identifying the effectors of the *P. aeruginosa* T6SS, including cell-wall-targeting enzymes (Type VI amidase effectors named Tae and Type VI glycoside hydrolase effectors named Tge), cell membrane-targeting enzymes (Type VI lipase effectors name Tle), nucleases, and NAD(P) glycohydrolase effectors. These effectors participate in inter- and intra-species competition, and they are produced concomitantly with specific immunity proteins that neutralize cognate toxins to prevent autointoxication (Russell et al., 2011, 2012, 2013; Whitney et al., 2015). TplE, a novel H2-T6SS-dependent lipase effector, was recently identified (Jiang et al., 2016). TplE is an antibacterial lipolytic toxin and TplEi interacts with TplE to provide protection from TplE (Jiang et al., 2016). The crystal structure of the TplE and TplEi complex provided structural insights into the mechanism of TplE functioning as an antibacterial lipolytic toxin and TplEi functioning as an immunity protein (Lu et al., 2014). Disrupting the interaction between TplE and TplEi could release the toxin activity and cause bacterial cell death, prompting us to target the TplE-TplEi interaction for the development of novel antimicrobial agents.

Herein, we solved the crystal structure of TplEi in complex with a TplE peptide (residues 82-108) generated by treatment with subtilisin. The TplE peptide (denoted the “L” peptide) has a high binding affinity for TplEi *in vitro* and precludes the formation of the TplE-TplEi complex, which in turn releases the toxicity of TplE and thereby induces bacterial cell death. This is the first time that the T6SS effector is targeted as an antibacterial candidate, providing a proof of concept for the use of “L” peptides as possible drug leads for combating *P. aeruginosa* infection.

## Materials and methods

### Construct design, protein expression and purification

Plasmid-encoding TplEi and/or TplEi-TplE complex (pETDuet-1 vector with ORF1 encoding TplEi and ORF2 coding TplE) was transformed into *E. coli* strain Rosetta™ (DE3) competent cells (Novagen) for expression. The bacterial cultures were grown in LB medium at 37°C. The induction was initiated by the addition of IPTG (0.2 mM for TplEi-TplE complex; 0.5 mM for TplEi alone) when the culture reached OD_600_ = 1.2. The bacterial culture was incubated with shaking at 25°C overnight after the induction. The bacterial cells were then harvested by centrifugation (5,000 rpm, 30 min) and re-suspended in lysis buffer containing 20 mM Tris-HCl pH 8.0, 150 mM NaCl, 10 mM imidazole and 4 mM β-mercaptoethanol and disrupted by ultrasonication on ice. The cell debris was removed by centrifugation at 13,000 rpm for 60 min. The clarified supernatant was loaded to Ni-NTA resin (Qiagen). The TplEi and/or TplEi-TplE complex was next loaded on a HiTrap Q HP column (GE Healthcare) and eluted with the linear gradient of 75–1,000 mM NaCl. Finally, the eluted proteins were concentrated and applied to a Superdex 200 HR 10/30 column (GE Healthcare) equilibrated with 20 mM Tris-HCl pH 8.0, 100 mM NaCl and 2 mM DTT. The selenomethionine-substituted TplEi-TplE complex was produced by expression in the *E. coli* methionine auxotrophic strain B834 (DE3) in LeMASTER medium containing L-selenomethionine. The purification procedure for the SeMet derivative was the same as that of the native protein.

### Crystallization and structure determination

The crystallization experiments were performed using the hanging drop vapor diffusion setup at 22°C by mixing 1 μl of buffer and 1 μl of protein solution equilibrated over a 0.3 ml reservoir solution. Crystallization trials of TplEi alone and TplEi-TplE complex were subjected to *in situ* limited proteolysis by incubating trace amounts of trypsin and subtilisin from the Proti-ACE Kit (Hampton Research). The SeMet-labeled TplEi were grown in 0.52 M lithium sulfate, 13% PEG8000 after 48 h of incubation. The TplEi-TplE complex crystal appeared 1 month later at 22°C. The optimized condition for the TplEi-TplE complex was achieved by mixing 1 μl of buffer containing 0.1 M sodium HEPES, 20% (w/v) PEG 10,000 and 1 μl of protein solution with trace amounts of subtilisin. Crystals were soaked in reservoir solution supplemented with 10% ethylene glycol for 30–60 s before flash freezing in liquid nitrogen. X-ray diffraction experiments were conducted at beam line BL17U in Shanghai Synchrotron Radiation Facility (SSRF). All diffraction data were processed with the XDS package (Kabsch, 2010). The software program AUTOSHARP/SHARP was used to locate the Se atoms and to calculate the initial phase, producing an interpretable electron density map (Bricogne et al., 2003). The atomic model was built manually using the program Coot (Emsley and Cowtan, 2004) and was refined using PHENIX (Adams et al., 2010). The final model has excellent refinement statistics and stereochemistry quality. All of the structure figures were prepared using PyMOL (http://www.pymol.org).

### Peptide synthesis

The “L” peptide (TplE residues 82-108) and its mutants (Table S1) derived from a complex crystal structure between TplEi and TplE (residues 82-108) were synthesized using a standard solid-phase FMOC N-(9-fluorenyl)methoxycarbonyl) method purchased from Scilight Biotechnology LLC, as previously described (Yao et al., 2012). All of the peptides were acetylated at the N-terminus and amidated at the C-terminus. Synthesized peptides were purified by reversed-phase high-performance liquid chromatography (HPLC) and verified for purity >98% and correct amino acid composition by mass spectrometry. The peptides were freeze-dried into powder and dissolved at 4 mg/ml according to the production report for the stock solution.

### Isothermal titration calorimetry

Isothermal titration calorimetry (ITC) assay was performed with a MicroCalTM iTC200 calorimeter (MicroCal, USA) at 25°C; both the protein and peptides were dissolved with the same buffer (20 mM Tris-HCl, pH = 8.0, 100 mM NaCl). The concentration of TplEi was between 0.03 and 0.04 mM. The concentrations of wild type peptides and mutants were between 1 and 2 mM. Titration was scheduled with 18 consecutive injections of 2 μl of peptide with a 120 s interval between injections, using a stirring rate of 600 rpm. Data acquisition and analysis were performed using MicroCal Origin software (version 7.0).

### Size-exclusion chromatography

Superdex 200 10/300GL column (GE Healthcare) was equilibrated with the buffer containing 20 mM Tris-HCl (pH 8.0) and 100 mM NaCl and calibrated using molecular weight standards, γ-globulin (158 kDa), ovalbumin (45 kDa), myoglobin (17 kDa), and vitamin B12 (1.35 kDa). The purified TplEi and/or TplEi-TplE complex (~1 mg/ml) was loaded onto the column running at a flowrate of 0.15 ml/min.

### Cell-toxicity assay

Cell-toxicity assay was carried as previously described with minor modification (Jiang et al., 2016). Briefly, *E. coli* BL21 (DE3) strains harboring plasmids expressing periplasmic-targeted TplE, TplE-TplEi and the “L” peptide were grown overnight and serially diluted in LB medium at 10-fold. A 2 μl bacterial dilution was spotted onto LB agar plate containing 0.045 mM IPTG. Images were taken after 24 h growth.

## Results

### Identification of a peptide from TplE that disrupts the TplE-TplEi complex

Immunity proteins specifically bind their cognate effectors, thereby neutralizing their hazardous activity, and disrupting the interaction between effectors and immunity proteins represents a potential antibacterial strategy. The goal of this study was to identify a peptide that can specifically bind to the protein interaction interface of TplEi to preclude the formation of the TplE-TplEi complex. The crystal structure of the TplE-TplEi complex (also known as the Tle4–Tli4 complex) was recently solved (Lu et al., 2014). However, the interaction between TplE and TplEi is mediated by numerous hydrophobic contacts, hydrogen bonds or salt-bridges, which involves a large collection of residues and several discrete regions of TplE. It is therefore difficult to predict which regions play a pivotal role in TplEi binding based on this structure.

**Figure 1:**
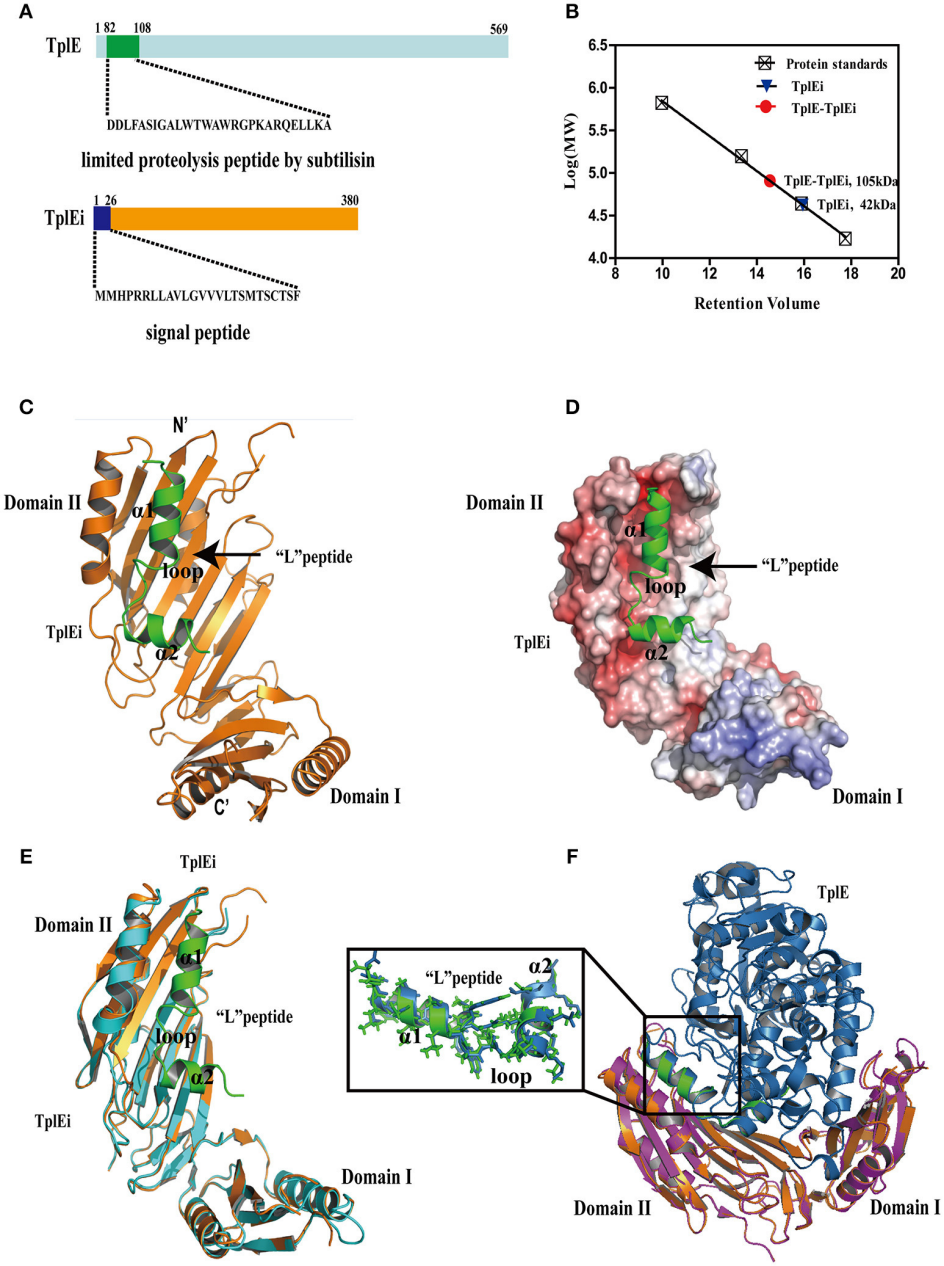
The overall structure of TplE peptide and TplEi complex. **(A)** Structure of the TplE and TplEi proteins. The sequence of TplE peptide digested by subtilisin and TplEi signal peptide are shown. **(B)** Molecular weight of TplEi and the TplE-TplEi complex assessed by size exclusion chromatography. The Superdex-200 HR 10/300GL column was equilibrated with protein standards γ-globulin 158 kDa, ovalbumin, 44 kDa, myoglobin17 kDa and vitamin B12, 1.35 kDa. The molecular weights of TplE and TplE-TplEi were calculated as 42 and 105 kDa, respectively. **(C)** Ribbon diagram representation of TplE peptide-TplEi complex. TplEi (orange) is shown in cartoon representation, and the TplE peptide (green) is also shown in a cartoon representation. **(D)** Surface electrostatic view of the TplE peptide-TplEi complex with their orientations corresponding to those shown in **(C)**. The peptide (D82-A108 in cartoon representation) binds in the hydrophobic groove of TplEi. **(E)** Superimposition of the crystal structures of TplEi alone and TplEi-TplE peptide. **(F)** Superimposition of the crystal structures of TplEi-TplE peptide and TplEi-TplE complex(PDB:4R1D).

To identify the region of TplE conferring the strongest binding of the immunity protein, we designed the experiment of *in situ* proteolysis and crystallization (Figure S1A). We first purified and concentrated the TplEi/TplE-TplEi complexes (Figure 1B), and incubated them with trypsin (~1:2,000 w/w) or subtilisin (~1:8,000 w/w) prior to crystallization trials. Because regions buried in tight protein-protein interfaces are more resistant to proteolysis than looser structural elements, proteases can digest all but the key fragments essential to the TplE-TplEi interaction, and these fragments can be identified by crystal structure determination. As anticipated, after 30 days of proteolysis and crystallization, we first observed crystals growing from the subtilisin-treated TplE-TplEi complex sample, and crystals diffracted the X rays to 2.2Å (Table 1). We determined the crystal structure using single-wavelength anomalous dispersion (SAD). Because we independently determined this structure before the releasing of Tle4–Tli4 full complex structure (PDB ID: 4R1D), the de novo structure determination using SAD was necessary. Surprisingly, the majority of TplEi was intact, whereas TplE had been degraded except for a 27 residue fragment that remained bound to TplEi (Figure 1A). The electron density for residues 82-108 of TplE is well defined in the final map apart from the head and tail region (Figure S1B). Next, we also obtained crystals of TplEi alone and determined the structure at 3.06 Å resolution using SAD. The TplEi apo structure is very similar with peptide-bound TplEi. Parameters of data collection, structure determination and refinement are summarized in Table 1.

**Table 1:** Data collection and refinement statistics.

	**TplEi (SeMet derivative) (PDB ID: 5H7Z)**	**TplEi-TplE (SeMet derivative) (PDB ID: 5H7Y)**
**DATA COLLECTION**		
Space group	I41	P6122
Cell dimensions		
a, b, c (Å)	182.23, 182.23, 78.87	113.61, 113.61, 128.13
α, β, γ (°)	90.00, 90.00, 90.00	90.00, 90.00, 120.00
X-ray source	SSRF BEAMLINE BL17U	SSRF BEAMLINE BL17U
Wavelength (Å)	0.9792 (Se peak)	0.9792 (Se peak)
Data range (Å)	31.25–3.06	34.13–2.20
Reflections unique	46,474	47,162
*R*^a^_sym_ (last shell)	0.624 (0.10)	0.575 (0.09)
*I*/σ*I* (last shell)	14.44 (2.67)	26.69 (5.51)
Completeness (%) (last shell)	96.9 (81.40)	99.8 (98.80)
Redundancy (last shell)	4.28 (3.34)	16.22 (15.00)
**REFINEMENT**		
Resolution range (Å)	31.25–3.06	34.13–2.20
Reflections (non-anomalous), cut-off, cross validation	47,977 (46,474), *F* > 1.34, 2,380	47,271 (47,162), *F* > 1.34, 2,397
*R*^b^_work_/*R*^c^_free_ (last shell)	0.2031/0.2448 (0.3308/0.3105)	0.1948/0.2244 (0.2574/0.2777)
**ATOMS**		
Non-hydrogen protein atoms	4,834	2,587
Protein	4,774	2,432
Ligands	0	0
Solvent	60	155
*B*-factors average (Å^2^)	70.16	38.97
Protein (Å^2^)	69.98	38.95
Ligands (Å^2^)	0	0
Solvent (Å^2^)	98.10	39.54
r.m.s.d		
Bond lengths (Å)	0.003	0.012
Bond angles (°)	0.872	1.188
**VALIDATION**		
Clash score, all atoms	4.43	5.42
% Poor rotamers	0.78	0.74
% residues in favored regions, allowed regions, and outliers in the Ramachandran plot	95.44, 4.56, 0	97.59, 2.41, 0

a *R_sym_ = $\underset{hkl}{\sum }$$\underset{j}{\sum }$|I_hkl, j_-I_hkl_|/$\underset{hkl}{\sum }$$\underset{j}{\sum }$I_hkl, j_, where I_hkl_ is the average of the symmetry-related observations of a unique reflection*.

b *R_work_ = $\underset{hkl}{\sum }$ ||F_obs_(hkl)|-|F_calc_(hkl)||/$\underset{hkl}{\sum }$|F_obs_(hkl)|*.

c *R_free_ = the cross-validation R factor for 5% of reflections against which the model was not refined*.

As shown in Figures 1C,D, residues 82-108 of TplE is comprised α1 and α2 helices that are connected by a disordered loop region. Because α1 is significantly longer than α2 and the two helices are nearly perpendicular to each other, the peptide exhibits a “L” shape, thus, we designated it as “L” peptide based on its shape. The “L” peptide accommodates a negatively charged groove of domain II of TplEi (Figure 1D). Using PDBePISA (http://www.ebi.ac.uk/msd-srv/prot_int/pistart.html), we calculated the buried area by “L” peptide (1173.6Å^2^), accounting for 46.4% of the total surface area of the peptide, and the ΔG value (the solvation free energy gain upon formation of the interface) was −19.6 kcal/mol. By comparison, the surface area buried by the intact TplE in the TplE-TplEi complex is 2,735.5 Å^2^, which is more than double that of the “L” peptide. However, the ΔG of the TplE-TplEi complex is −21.4 kcal/mol, which is not significantly different from that of the TplEi-“L” peptide complex. These results suggest that the region corresponding to the “L” peptide is crucial for the TplE-TplEi interaction, which is consistent with our proteolysis and crystallographic results.

The structure of TplEi alone is very similar to that observed in the peptide-bound form (Figure 1E), with only a handful of side chains with different rotamer conformations (Figure S1C). Specifically, the side chains of residues such as Mse274 move out to avoid clashes, F230 and R209 are closer to the peptide, and residues E219, E211, and K266 change their side chain conformations to form hydrogen bonds or salt bridges with the peptide. The similarity between the free and peptide-bound forms suggests that the TplEi structure is highly compact and rigid, and the “L” peptide binds via a structurally stable pocket on TplEi. We also superimposed the structures of TplEi-“L” peptide complex with TplE-TplEi complex (PDB:4R1D) (Figure 1F). While the structures TplEi are well superimposed, the “L” peptide overlays with the protein interaction interface of the small subdomain of the TplE Cap domain (Figure 1F). The structural superimposition of the “L” peptide and TplE aligned 25 Cα atoms with a rmsd value of 0.59Å, indicating that the conformation of the “L” peptide alone is nearly identical to its conformation in the context of the full protein (Figure 1F). Importantly, the mode of interaction between the region corresponding to the “L” peptide and TplEi remains unchanged in the TplE-TplEi complex.

### TplE peptide has high binding affinity to TplEi

**Figure 2:**
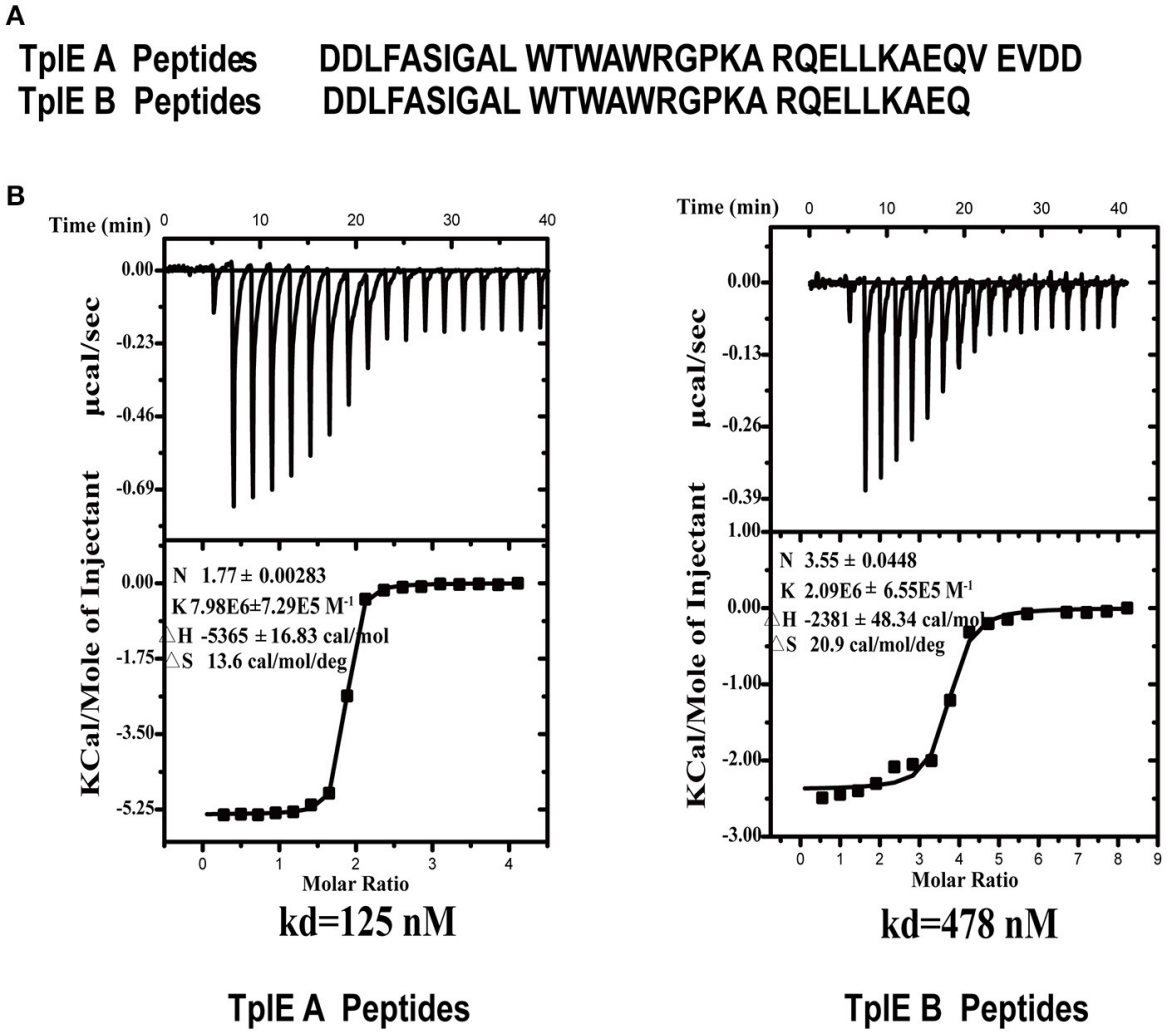
ITC analysis of the interaction between the TplE peptide and TplEi. **(A)** Sequences of A peptides and B peptides derived from TplE peptide crystal structure. B peptides add EQ residues, and A peptides add EQVEVDD residues. **(B)** The binding affinities of TplE peptide (A peptides and B peptides) to TplEi were determined using ITC. The upper part of each panel shows the raw data (top) (thermal power against time), and the bottom part of each panel shows the binding isotherm (normalized heat against molar ratio of reactants) for each injection. The data were fitted to a single-site binding model. The dissociation constants (K_d_) are reported at the lower panels. The number of binding sites (N), enthalpy (ΔH), and entropy (ΔS) is also provided.

The crystal structure of TplE peptide-bound TplEi prompted us to speculate that the TplE “L” peptide may have high binding affinity to TplEi and could be a potential inhibitor targeting TplE/TplEi complex. To test our hypothesis, we synthesized two types of “L” peptide: A peptides and B peptides (Figure 2A). Using isothermal titration calorimetry (ITC), we measured a dissociation constant (*K*_*d*_) between A/B peptides and TplEi and evaluated their binding affinity *in vitro*. Intriguingly, while the dissociation constant (*K*_*d*_) is ~125 nM between the A peptides and TplEi the binding affinity of the shorter B peptides decreased with a *K*_*d*_ = 478 nM (Figure 2B), therefore only A peptides were used in the following characterizations. Comparing with B peptides, A peptides contain an additional C-terminal _111_VEVDD_115_ segment. This segment was not observed in the structure of “L” peptide-TplEi complex, indicating that this region does not bind TplEi. The structure of TplE-TplEi complex reported by Lu et al. also shows that _111_VEVDD_115_ segment is not in contact with the immunity protein. Therefore, the contribution of _111_VEVDD_115_ segment in binding affinity was unlikely to increase the binding interface. Rather, the original purpose of adding the _111_VEVDD_115_ segment was to increase charging at the C-terminus of the peptide, therefore it may improve the stability and overall folding of the peptide. The increased binding affinity of A peptides over B peptides seem to support our hypothesis. Collectively, the ITC data suggests that the “L” peptide could occup the binding interface on TplEi, therefore it is possible that the “L” peptide acts to prevent TplE/TplEi complex formation.

### Details of TplE “L” peptide-TplEi interaction and validation by mutagenesis studies

**Figure 3:**
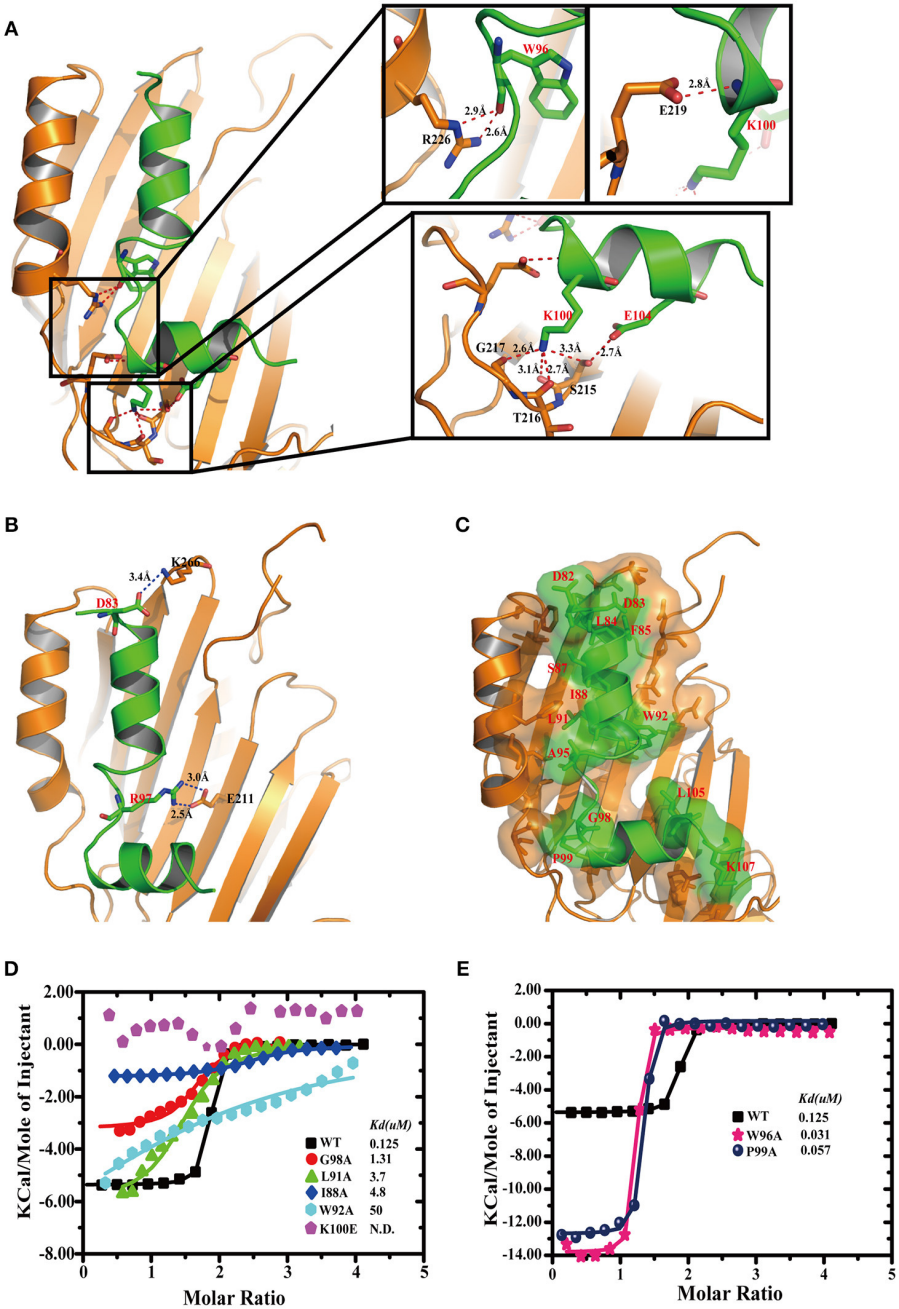
Details of TplE peptide recognition by TplEi and mutagenesis studies. **(A)** Details of the interaction between the TplE peptide and TplEi are shown in stereo view. The side chains of the residues involved in the interdomain are illustrated in the stick model. (Right) Close-up views of hydrogen bonding interactions are highlighted by dashed lines in red. **(B)** Salt bridge interactions are highlighted by dashed lines in blue. **(C)** Hydrophobic interactions are represented by transparent surfaces. Distribution of hydrophobic residues (red) on the surface. The hydrophobic interaction is generated by the LigPlot^+^ program and is plotted by PyMol. **(D)** Representative of ITC experiments of TplE peptide and its mutants. Structure-guided mutations prevent or reduce binding to TplEi. ITC binding curves comparing binding of TplE peptide mutants with TplEi. **(E)** Mutations W96 and P99 increase the TplE peptide-TplEi interaction. ITC binding curves for indicated TplE peptide mutants with TplEi.

Details of the TplE “L” peptide interaction with TplEi involves two salt bridges, eight hydrogen bonds and 147 non-bonded contacts through PDBsum analysis (de Beer et al., 2014) (Figures S2A,B). The “L” peptide adopts a highly extended conformation except for P99 introducing a turn in the C-terminus. The “L” peptide lying across the TplEi domain II area formed a large hydrophobic pocket. As illustrated in Figure 3A, approximately eight direct hydrogen bonds were found between TplEi and the “L” peptide (red dashes). In particular, “L” peptide residue K100 is anchored in the deep pocket (Figures 1D, 3A) through four hydrogen bonds formed with TplEi residues S215, T216, and G217. Three hydrogen bonds are formed between K100 side chain and the carbonyl groups of TplEi residues, and one hydrogen bond is formed between K100 side chain and S215 side chain. We validated the hydrogen bonding contribution by ITC, and the results showed that K100E mutation completely abolished the “L” peptide binding (Figure 3D), stressing the importance of K100 in the “L” peptide binding ability. Two salt bridges were also observed between TplEi and the “L” peptide (blue dashes) (Figure 3B). The side chain of K266 and the side chain of E211 in TplEi are connected to the “L” peptide through two salt bridges with the side chain of residue D83 and the side chain R97 of the “L” peptide, respectively. However, mutation in “L” peptides R97 to opposite charges has no effect on “L” peptide binding (Figure S2C). This result suggests that the salt bridge might not have significant effect on binding ability. TplEi is assembled into a highly rigid structure, not only in the apo but also in the peptide-bound structure. “L” peptide residues D82, D83, L84, F85, S87, I88, L91, W92, A95, G98, P99, L105, and K107 are buried in TplEi surface areas (Figure 3C). To validate the contribution of the hydrophobic interaction to “L” peptide recognition, we performed ITC assay. We found that alanine substitutions of key amino acids in the “L” peptide decreased binding to TplEi, especially for W92A mutation, resulting in a 500-folds reduction in binding affinity. Compared with salt bridge interaction, the hydrophobic interaction plays more important role in “L” peptide binding (Figure 3D). However, two mutations (W96A and P99A) increased binding affinity compared to WT (Figure 3E); this phenomenon can be explained by the fact that W96 backbone atoms does not affect hydrogen bond formation to TplEi R226, and aromatic group mutation reduced steric hindrance. In contrast, P99A mutation eliminates a turn in the peptide and therefore increases binding affinity. This implies that optimizing the design of the “L” peptide can increase its binding ability.

It is surprising to find that a single mutation (like K100E) of the “L” peptide could abolish the binding with TplEi, regardless that a large area of the peptide is involved in the interaction with the immunity protein. To further elucidate the mechanism underlying the “L” peptide binding, we mutated several key residues of TplEi interacting with the “L” peptide, and measured the binding affinity of the “L” peptide to these TplEi mutants using ITC. The side chain of K100 of the “L” peptide forms four hydrogen bonds with TplEi, among which only one involves side chain interaction with S215 of TplEi, whereas the other three involve backbone atoms. Therefore, we introduced S215A mutation to TplEi, and found that the “L” peptide bound the S215A mutant with the Kd = 7.8 μM, indicating nearly 62-folds reduced binding affinity comparing to the affinity to the wild type TplEi (Kd = 0.125 μM, Figure S3). This data demonstrated that the disruption of a single hydrogen bond could indeed significantly disrupt the interaction of the “L” peptide and TplEi. Similarly, we prepared TplEi mutants E219A and R266A, respectively. E219 forms a hydrogen bond with backbone NH group of K100 of the “L” peptide, and R266 forms two hydrogen bonds with the carbonyl group of W96 of the peptide (Figure 3A). As expected, the binding affinities of the “L” peptide with E219A and R266A mutants reduced significantly. The *K*_*d*_ for E219A and R266A were 35.5 and 46.9 μM, respectively, indicating 284- and 375-folds decreased binding affinities (Figure S3).

### The “L” peptide displaces the TplE effector activator to induce autointoxication

**Figure 4:**
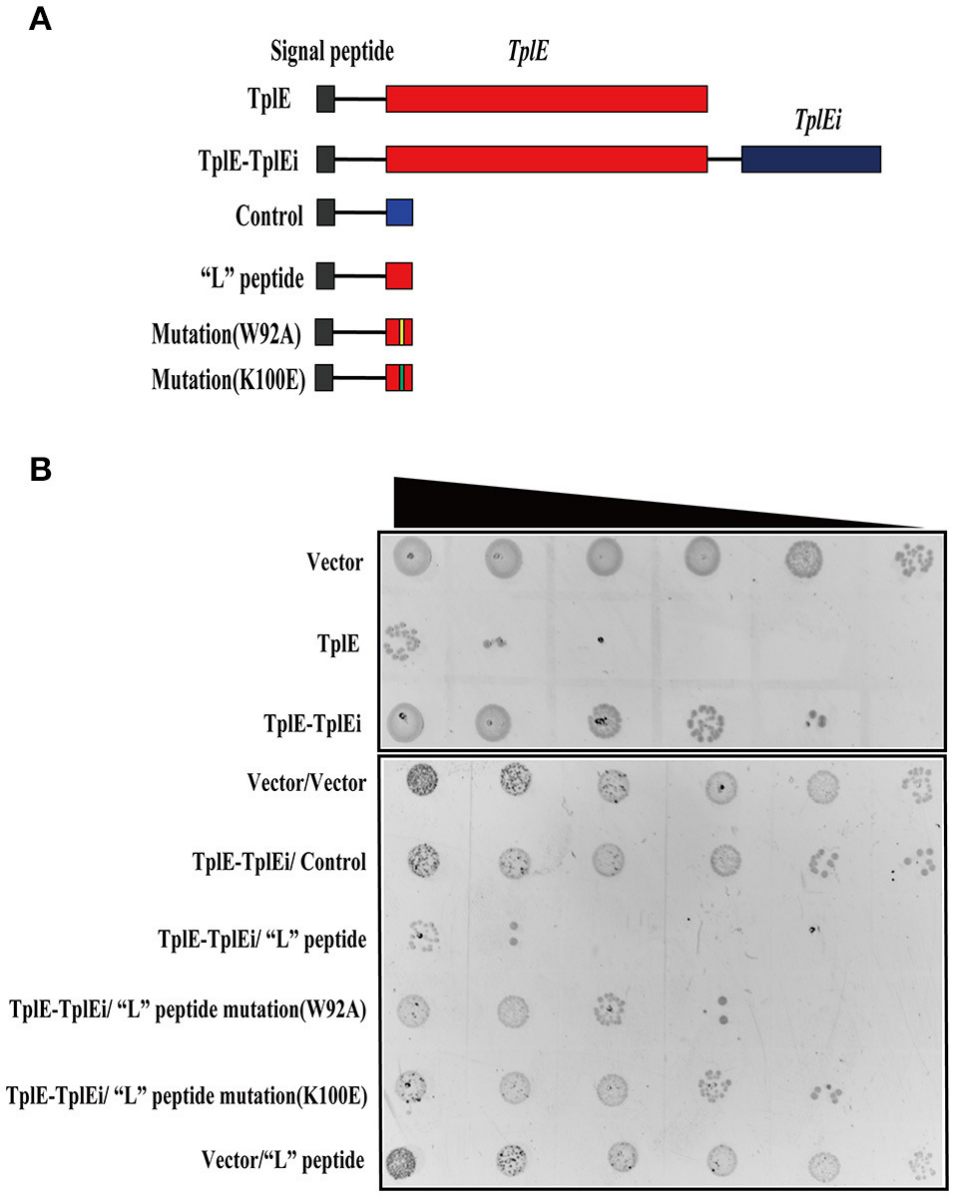
“L” peptide activates the TplE toxin by competing with TplEi. **(A)** Diagrams of TplE, TplE-TplEi and “L” peptide expressing constructs. Expression of vector sequence and “L” peptide mutant with the PelB signal peptide was used as a control. **(B)** Growth of *E. coli* strain BL21 (DE3) expressing with the constructs showed in **(A)** on LB agar plates. A 10-fold serial dilution of overnight culture was spotted on LB agar plates from left to right. The experiments were performed at least 3 times.

As the “L” peptide has high affinity and specificity to TplEi *in vitro*, we hypothesized that it could function as an activator of TplE by competitive binding to TplEi, which could in turn induce bacterial death *in vivo*. To test this hypothesis, we performed *Escherichia coli* cell toxicity assays as previously described (Dong et al., 2013; Jiang et al., 2016). Consistent with the previous report that TplE is an antibacterial lipolytic toxin and TplEi is a cognate periplasmic immunity protein (Jiang et al., 2016), periplasmic expression of TplE in *E.coli* resulted in a significant inhibition of growth, while co-expression of TplEi repressed the TplE-dependent growth inhibition (Jiang et al., 2016). The role of “L” peptide was further investigated *in vivo* using this method. As expected, expression of the “L” peptide but not a control peptide resulted in significant TplE-dependent growth inhibition (Figures 4A,B). Consistent with the *in vitro* data that K100E mutation abolishes and W92A weakens the binding of “L” peptide with TplEi (Figure 3D), expression of the K100E mutated “L” peptide resulted in a strong and expression of another mutated “L” peptide (W92A) resulted in a moderate decreased growth inhibition. Taken together, these results suggested that the “L” peptide could disrupt the TplE-TplEi interaction to release TplE toxicity.

## Discussion

**Figure 5:**
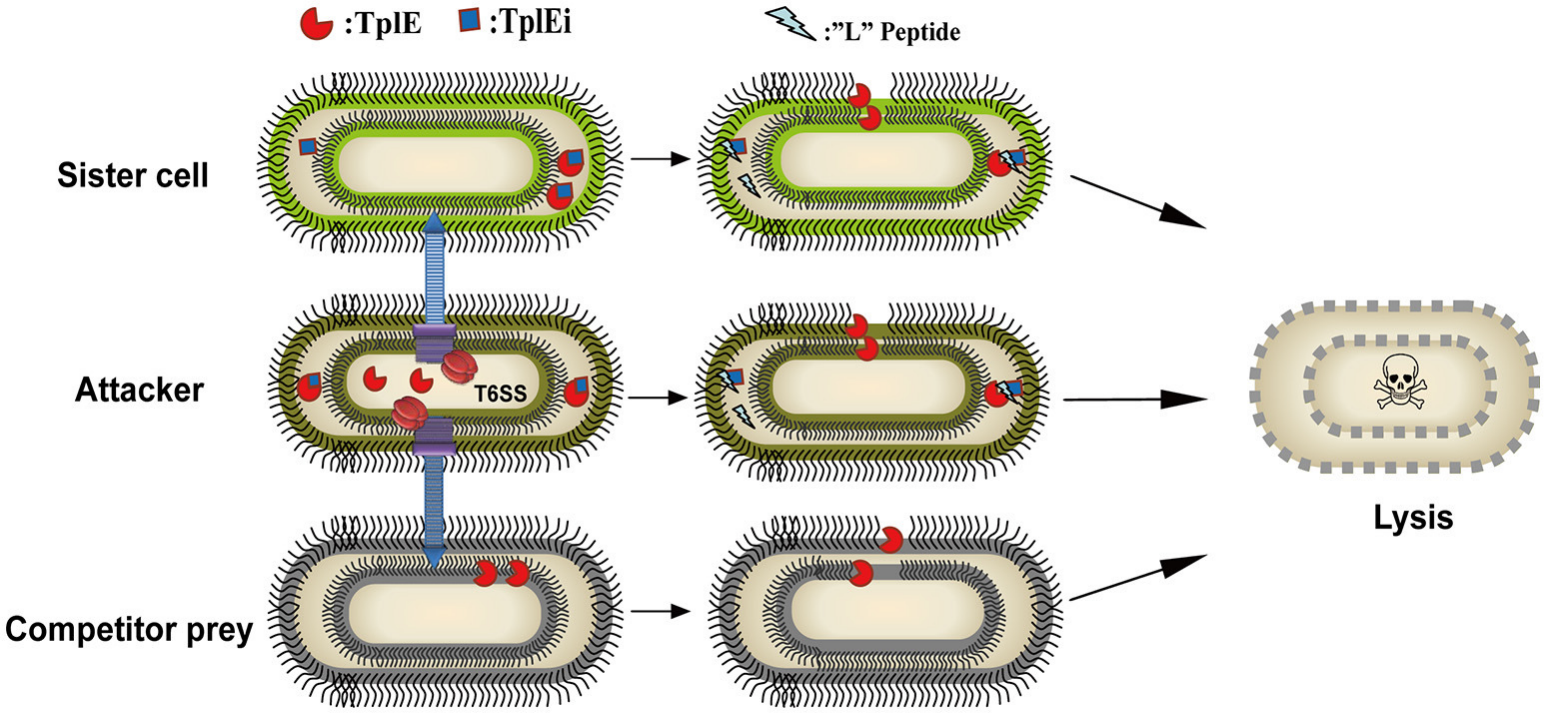
Model of a proof of concept of the TplE peptide. The schematic depicts the TplE peptide disrupting the TplE-TplEi complex and activating the TplE toxin to induce bacterial autolysis. TplE and TplEi are shown as crescents and squares, respectively, and the TplE peptides are shown as zigzags. In attacker cells (brown oval), TplE is neutralized by TplEi in its periplasm to protect itself from suicide. When an attacker cell delivers the TplE to sister cells (chartreuse oval) periplasm, TplEi protein is employed to protect the sister cell from fratricide. When TplE is injected into the periplasm of competitor prey lacking TplEi, TplE directly targets the bacterial membrane (black curve). When the TplE “L” peptide is administered to the attacker cell and/or sister cell, it directly activates the TplE toxin by disrupting the preformed TplE-TplEi complex or preventing complex formation. Once TplE is activated, it will degrade self-membranes and finally induce suicide or fratricide.

The T6SS has recently received much attention because it plays an important role in intra-and inter-species completion between bacteria, and hence in microbial pathogenesis (Durand et al., 2014; Russell et al., 2014; Alcoforado Diniz et al., 2015). TplE, a new T6SS trans-kingdom toxin, targets the conserved and essential cell membranes of rival bacteria to cause bacteriolysis, while TplEi functions as a cognate immunity protein to prevent self-intoxication (Jiang et al., 2016). It would be a powerful antibacterial strategy to release the TplE activity by using small compounds or peptides as inhibitors of the TplE-TplEi interactions. In this study, we identified a TplE “L” peptide upon protease-assisted co-crystallization with TplEi. The TplE “L” peptide displays strong binding affinity to the TplEi protein (Figure 2B), and further analysis showed that binding to TplEi might disrupt the TplEi-TplE interaction and unleash the TplE toxin to induce bacterial autointoxication and autolysis (Figure 5).

The structure of the TplE-TplEi complex reveals that the small subdomain of the TplE Cap domain interacts with domain II of TplEi, whereas the large subdomain of the TplE Cap domain interacts with domain I of TplEi (Lu et al., 2014). Our combined proteolytic and crystallographic experiments showed that the “L” peptide binds specifically to a groove in domain II of TplEi. Our calculations showed that formation of the TplE-TplEi and TplEi-“L” peptide complexes proceeds with similar ΔG values, suggesting that the interaction between the “L” peptide and TplEi plays a major role in the formation of the TplE-TplEi complex. This is consistent with the previous report that the groove in domain II of TplEi is important for TplE-TplEi complex formation (Lu et al., 2014). Thus, the occupation of this groove by the “L” peptide might disrupt the TplE-TplEi interaction and release TplE lipase activity.

The targeting of T6SS effector-immunity (E-I) pairs is analogous to toxin-antitoxin (TA) systems, the exploitation of which has received considerable attention as a strategy for developing antibacterial drugs (Lee and Lee, 2016; Kang et al., 2017). For example, several peptide inhibitors based on the *Bacillus anthracis* PemIK interaction were designed to mimic the antitoxin and release the PemK toxin to kill the bacteria (Agarwal et al., 2010; Barbosa et al., 2012). A naturally occurring quorum sensing (QS) pentapeptide EDF from the *E. coli* MazE-MazF TA system competitively interacts with the MazE-binding site for MazF, and thereby overcomes the inhibitory activity of the antitoxin MazE, releasing the MazF toxin and ultimately causing cell death(Belitsky et al., 2011). Based on the structure of the VapB-VapC complex in *Mycobacterium tuberculosis*, several kinds of peptides mimicking the toxin and antitoxin were designed to disrupt the TA interaction and activate the ribonuclease activity of the VapC toxin (Lee et al., 2015; Kang et al., 2017), providing a novel antibacterial approach to the development of new antibiotics.

The TplE “L” peptide is an example of a toxin-mimicking peptide that binds to TplEi and disrupts the TplE-TplEi interaction, releasing the active TplE toxin. However, further work is needed to assess the potential of the “L” peptide for treating *P. aeruginosa* infections. For example, we only tested the efficacy of the “L” peptide against *E. coli*, and whether the expression of the peptide would affect the growth of *P. aeruginosa* still needs to be verified. In addition, the TplE “L” peptide must access the periplasm to disrupt the TplE-TplEi interaction, which could prove difficult, although cell-penetrating peptides (CPPs) attached to the TplE “L” peptide could potentially overcome this drawback to provide a novel therapeutic agent (Fosgerau and Hoffmann, 2015). Nevertheless, the present study provides a structural and biochemical basis for the development of antibacterial peptides, and provides a proof of concept for structure-based design and targeting of the T6SS in *P. aeruginosa* and other bacteria. The TplE “L” peptide represents a novel drug candidate for a T6SS-based therapeutic strategy against *P. aeruginosa*.

## Accession codes

The atomic coordinates and structure factors have been deposited in the Protein Data Bank with the accession codes 5H7Z for TplE and 5H7Y for TplE peptide-TplEi.

## Author contributions

SC, YS, and XG designed the study. SC, YS, and XG wrote the paper. XG and ZM purified and crystallized protein and determined structure. XG and ZM performed and analyzed ITC assay, Cell-toxicity assay. BQ designed construct for expression of protein. All authors reviewed the results and approved the final version of the manuscript.

### Conflict of interest statement

The authors declare that the research was conducted in the absence of any commercial or financial relationships that could be construed as a potential conflict of interest.
