# Supplementary material for: Structure-Based Prototype Peptides Targeting the Pseudomonas aeruginosa Type VI Secretion System Effector as a Novel Antibacterial Strategy
Source: Front Cell Infect Microbiol. 2017 Sep 20;7:411. doi: 10.3389/fcimb.2017.00411 (PMC5611513; doi:10.3389/fcimb.2017.00411)
Supplement: Supplementary file 1 [file DataSheet1.docx]

***Supplementary Material***

**Structure-based Prototype Peptides Targeting the *Pseudomonas aeruginosa* Type VI Secretion System Effector as a** **Novel Antibacterial Strategy**

Xiaopan Gao^1‡^, Zhixia Mu^1‡^, Bo Qin^1^, YiCheng Sun^1*^ and Sheng Cui^1*^

^‡^These authors contributed equally to this work.

^*^Correspondence:

Corresponding author mailing address:

Sheng Cui, PhD,

Institute of Pathogen Biology, Chinese Academy of Medical Science

No.9 Dong Dan San Tiao, Dong Cheng Qu100730, Beijing P.R. CHINA

Email: cui.sheng@ipb.pumc.edu.cn (S.C.)

YiCheng Sun, PhD,

Institute of Pathogen Biology, Chinese Academy of Medical Science

No.9 Dong Dan San Tiao, Dong Cheng Qu100730, Beijing P.R. CHINA

Email: [sunyc@ipbcams.ac.cn](mailto:sunyc@ipbcams.ac.cn) (Y.S.).

**SUPPLEMENTAL FIGURES AND TABLES**

**SI Figures 1**


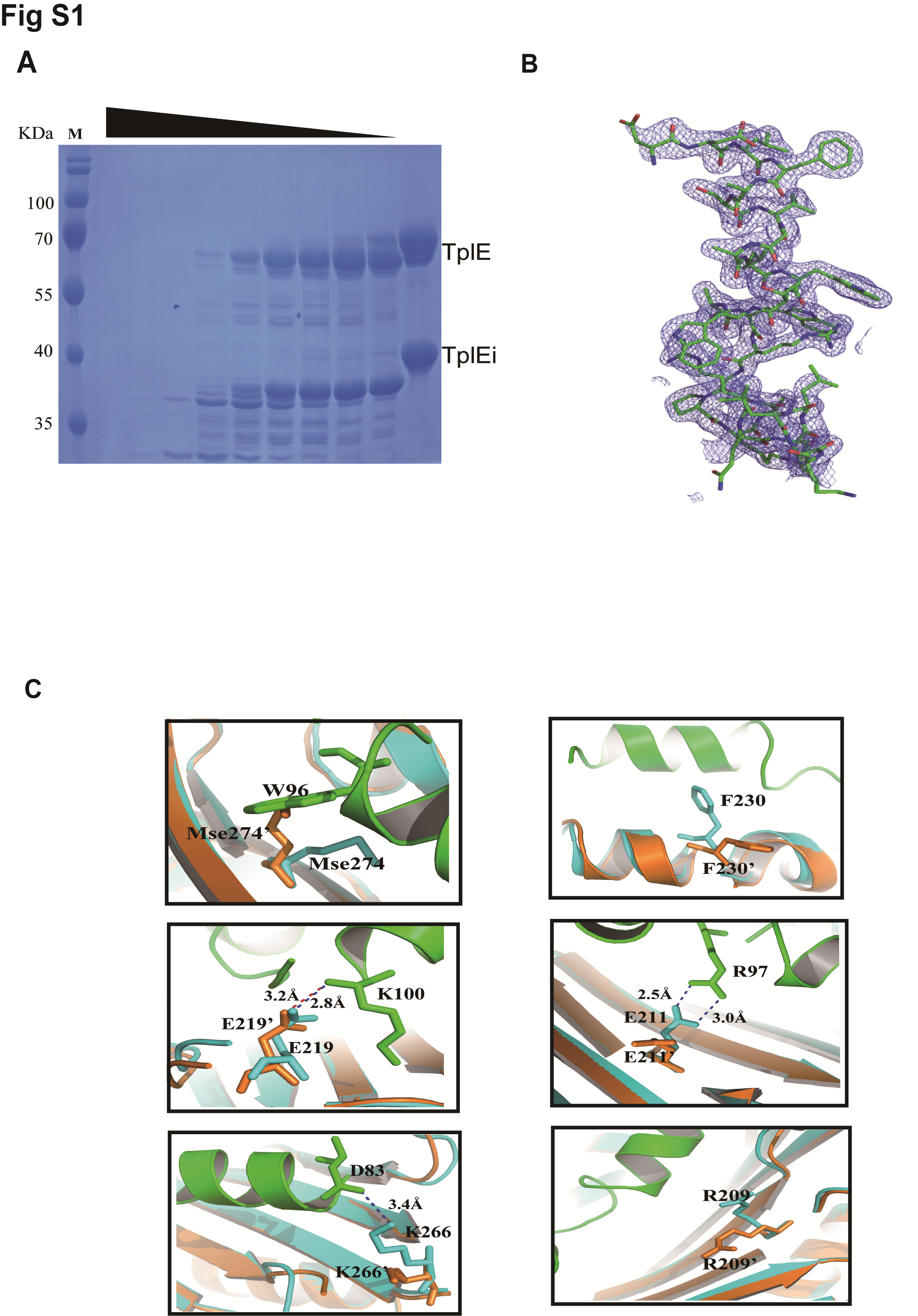


**Figure S1,Structure comparison of the TplEi with TplE peptide-TplEi complex**

(A)TplE-TplEi complex proteolytic digestion by subtilisin. In the right lane is untreated protein complex. Following lanes consist of 2-fold serial dilutions subtilisin concentrations from left to right*.* (B) Close-up view of a fragment that remained bound to TplEi. The peptide is shown with the superimposed final 2Fo-Fc electron density at contour level 1.2σ. (C) Side-chains conformations changes induced by TplE peptide binding.

**SI Figures 2**


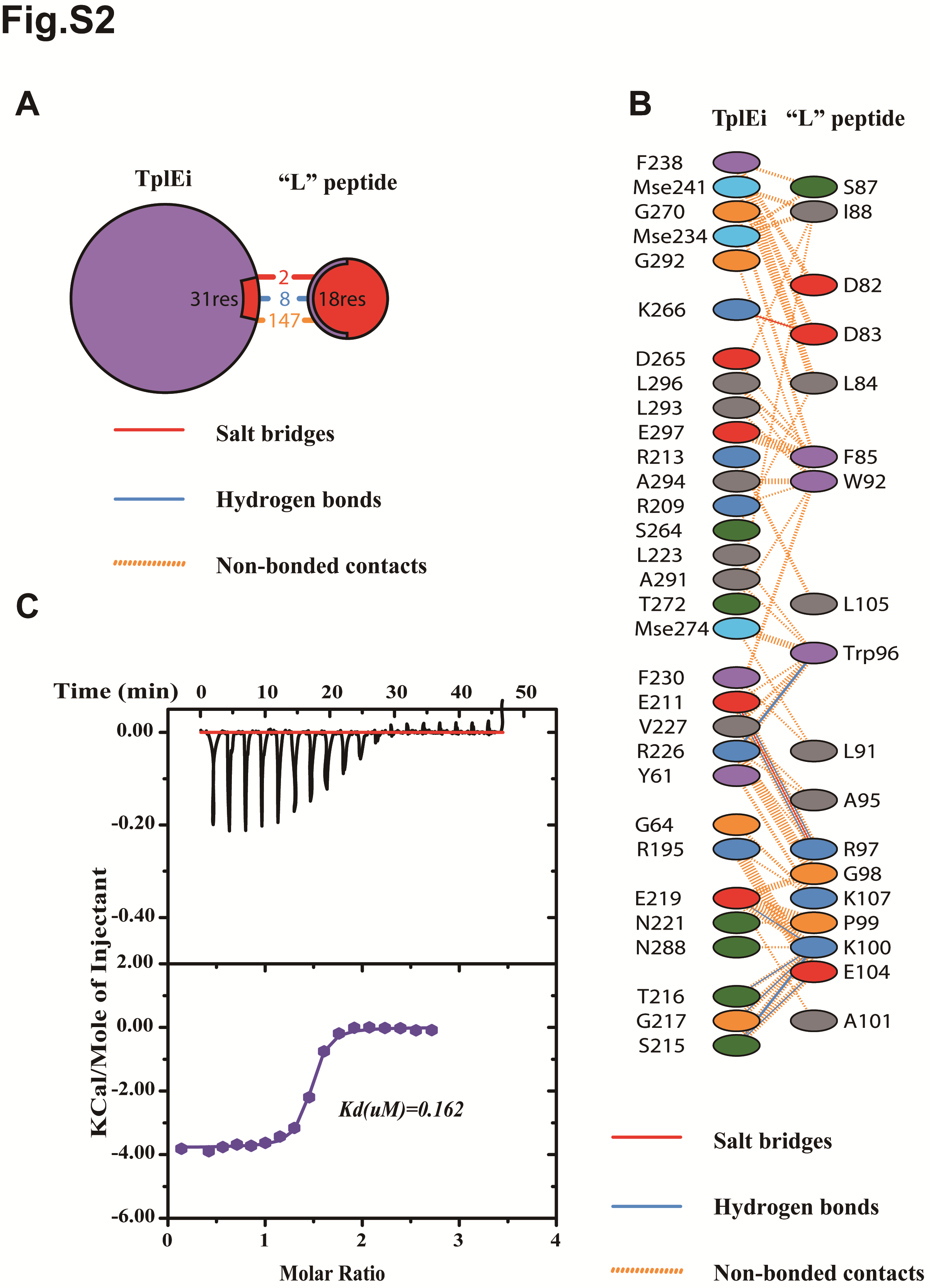


**Figure S2, Detail interactions analysis between TplEi and TplE peptide by PDB*sum***

(A)Schematic diagram of interactions beween protein chains. Interacting chains are joined by coloured lines, each representing a different type of interaction. (B)The H-bonds, salt bridges and non-bonded contacts are listed in detail. (C)ITC-based measurements of the salt bridges associated mutation to TplEi. Related Figure 3.

**SI Figure 3**


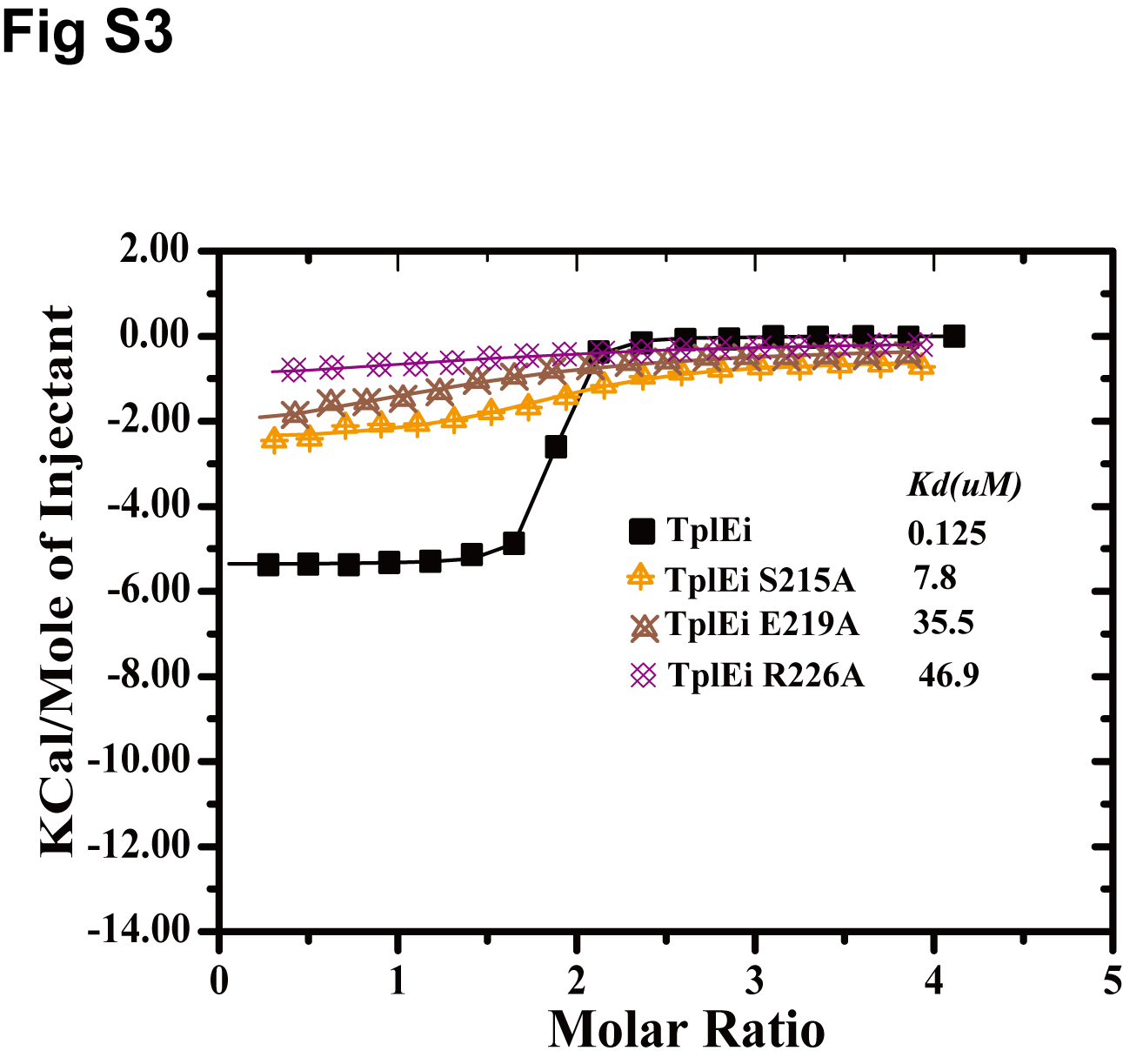


**Figure S3, ITC-based measurements of TplEi mutation to peptide**

**Tables**

**Table 1. Amino acid sequence of the peptides used is this study**

| Peptide | Sequence |
| --- | --- |
| A Peptides | DDLFASIGAL WTWAWRGPKA RQELLKAEQV EVDD |
| B Peptides | DDLFASIGAL WTWAWRGPKA RQELLKAEQ |
| Mutant based on A peptides(K100E) | DDLFASIGALWTWAWRGPEARQELLKAEQVEVDD K100E |
| Mutant based on A peptides (P99A) | DDLFASIGALWTWAWRGAKARQELLKAEQVEVDD P99A |
| Mutant based on A peptides (G98A) | DDLFASIGALWTWAWRAPKARQELLKAEQVEVDD G98A |
| Mutant based on A peptides (W92A) | DDLFASIGALATWAWRGPKARQELLKAEQVEVDD W92A |
| Mutant based on A peptides (W96A) | DDLFASIGALWTWAARGPKARQELLKAEQVEVDD W96A |
| Mutant based on A peptides (I88A) | DDLFASAGALWTWAWRGPKARQELLKAEQVEVDD I88A |
| Mutant based on A peptides (L91A) | DDLFASIGAAWTWAWRGPKARQELLKAEQVEVDD L91A |

Mutation of the residue is highlighted with red.
